# Desulfurization of Cu–Fe Alloy Obtained from Copper Slag and the Effect on Form of Copper in Alloy

**DOI:** 10.3390/ma15155110

**Published:** 2022-07-22

**Authors:** Baojing Zhang, Peizhong Feng, Tingan Zhang

**Affiliations:** 1 School of Materials Science and Physics, China University of Mining and Technology, No. 1, Daxue Road, Xuzhou 221116, China; zhangbj@cumt.edu.cn; 2 School of Metallurgy, Northeastern University, No. 3–11, Wenhua Road, Heping District, Shenyang 110819, China; zhangta@smm.neu.edu.cn

**Keywords:** desulfurization, copper slag, Cu–Fe, Fe–Mn, CaC_2_, nano-copper spheres

## Abstract

In order to realize the high-value utilization of copper slag, a process for preparing Cu–Fe alloy through the reduction of copper slag is proposed. The sulfur in the alloy exists in the form of matte inclusions, which is different from sulfur in molten iron. The reaction of CaO with Cu_2_S is difficult. It is necessary to add a reducing agent to promote desulfurization. To avoid the introduction of other elements, Fe–Mn and CaC_2_ additions were used as desulfurizers for the desulfurization of Cu–Fe alloy. The thermodynamics of the desulfurization reaction were calculated and the experimental process was studied. It was found that the Gibbs free energy of desulfurization reactions was negative for Fe–Mn and that CaC_2_ can reduce the sulfur in the alloy to 0.0013% and 0.0079%, respectively. The desulfurization process affected the shape of copper in the alloy. Part of copper in this alloy exists in the form of nano-copper spheres, and the size of the spheres is found to increase after desulfurization. Reducing agents can facilitate the desulfurization process of stable sulfides.

## 1. Introduction

Empirical experience has shown that 1 ton of copper will generate approximately 2–3 tons of copper smelting slag [1,2]. Global copper slag production amounts to 30 million tons [1,2]. Such a large amount of copper slag has put huge pressure on the environment, resources and energy. 

Copper slag is a valuable secondary resource, and contains about 40% iron and 0.3–5% copper [3,4,5,6]. At present, the main utilization method of copper slag is to extract the copper by flotation, extract the iron by reduction or magnetic separation, and then prepare the building materials [7,8,9,10]. Erdenebold et al. investigated the recovery of iron from copper slag [11,12,13]. Busolic et al. obtained Cu–Fe alloy from copper flash slag, with the aim to reduce the Cu content and obtain iron [14]. Heo et al. recovered iron from slag and the Fe recovery was about 90 wt.% [15]. Zhang et al. studied the reduction of the oxide system of iron and copper using hydrogen, with no investigation into metal recovery [16]. Few researchers focus on both copper and iron extraction. 

Copper is inevitably mixed into the iron extracted by reduction, and copper in iron will cause hot brittleness during the heat treatment process. The separation of copper and iron is difficult, which brings great difficulties to subsequent processing. Our research group proposed a new craft for the preparation of Cu–Fe alloy by reducing copper slag [1,2]. Copper and iron can be used as raw materials to make copper-containing steel or copper-containing cast iron, and copper and iron in slag are comprehensively utilized [17,18]. In the copper smelting process, copper slag is produced in the process of preparing matte. Therefore, the main form of copper in slag is mixed matte, and the main form is Cu_2_S [18]. It is necessary to study the desulfurization process for the application of Cu–Fe alloy.

At present, molten iron desulfurizers mainly include calcium desulfurizers, such as CaO, CaC_2_ and CaCO_3_, and metal desulfurizers, such as Mg, Al, and Mn. Freismuth used CaC_2_ to desulfurize pig iron, showing a good desulfurization effect [19]. Vaynman et al. studied Cu–Fe–Mn-based high-strength low-carbon ferritic steel [20]. S in Cu–Fe alloy obtained from copper slag mainly exists in the form of Cu_2_S, which is more stable and has a lower Gibbs free energy than FeS. The sulfur in molten iron can generate calcium sulfide by adjusting the alkalinity of slag or adding calcium oxide. However, Cu_2_S is difficult to react with CaO. According to the calculation results of thermodynamics, the Gibbs free energy of the reaction between Cu_2_S and CaO is greater than zero. 

Reducing conditions can promote the desulfurization reaction. Metal reductants introduce other elements during desulfurization, which affect the utilization of Cu–Fe alloy. Cu–Fe alloy can be used to prepare copper-containing antibacterial stainless steel or copper-containing high-chromium wear-resistant cast iron. A certain manganese content is required. CaC_2_ can be decomposed into calcium vapor and carbon at high temperature. Manganese and calcium, as reducing agents, can accelerate the desulfurization process. 

According to the different components of the target product, the metal desulfurizer can obtain the corresponding alloy products in the desulfurization process. After thoroughly researching the literature, we have not found similar studies using either Fe–Mn alloy or the CaC_2_ as desulfurization agents of Cu–Fe alloy. 

## 2. Experiment

### 2.1. Materials

**Figure 1:**
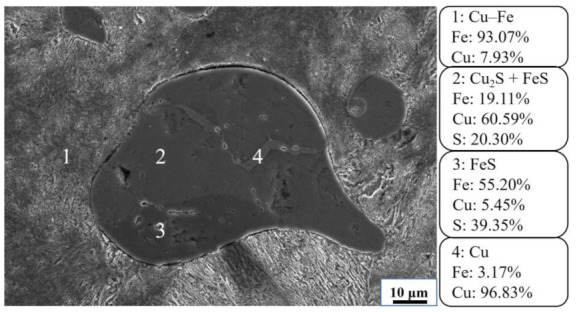
Electron microscope of Cu–Fe alloy.

The Cu–Fe alloy and slag used in this experiment were obtained via the reduction of copper slag, and their chemical compositions are shown in Table 1. The SEM micrograph and EDS results of the Cu–Fe alloy used in the experiment are shown in Figure 1 [21,22]. From the electron microscope, it can be seen that the main matrix of the metal is copper–iron-based alloy, which exists in the form of pearlite. The sulfur content in the metal is high, which is 1.32% and exists in the form of FeS and Cu_2_S. In Figure 1, the gray-white area is the Cu accumulation area (4), the gray area is the inclusion of FeS and Cu_2_S (2), and the gray-black area is the FeS accumulation area (3). The desulfurization agents are Fe–65 wt.% Mn (from Anyang manufacturer) and CaC_2_ (from Tianjin manufacturer).

### 2.2. Experimental Procedures

**Figure 2:**
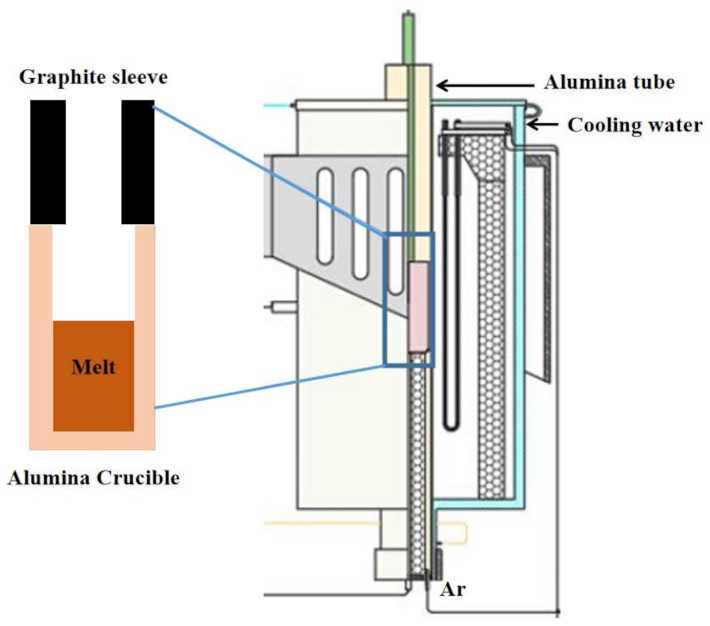
Experimental device.

**Figure 3:**
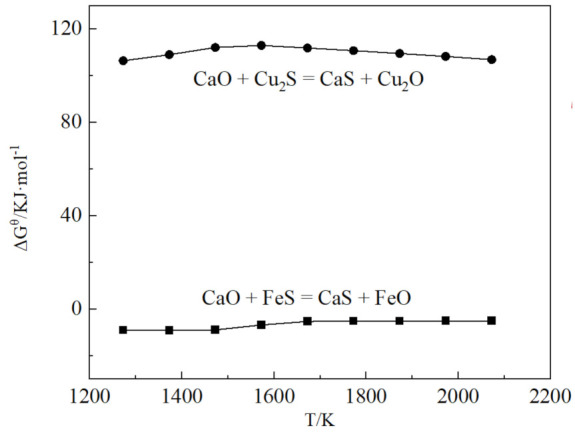
The Gibbs free energy of CaO desulfurization reaction.

The desulfurization experiment was carried out in a resistance furnace. The schematic diagram is shown in Figure 2. The reaction was carried out in an alumina crucible. The upper part of the alumina crucible was covered with a graphite sleeve to prevent the liquid level from rising and overflowing, which brought safety hazards. In total, 40 g of Cu–Fe alloy, 34 g of tailings and 15 g of CaO were added into the alumina crucible. Only CaO made it difficult to remove S from Cu–Fe alloy. The Gibbs free energy of the desulfurization reaction of CaO is shown in Figure 3. CaO can react with FeS, but it is difficult to react with Cu_2_S. Calcium oxide was used to adjust the alkalinity to ensure that it was above 2.0, which is helpful for desulfurization. Corresponding amounts of ferromanganese and calcium oxide were added as desulfurizers. The set temperature was 1698 K. After the temperature was raised to 1698 K, the temperature was maintained for 2 h, and then cooled to room temperature with the furnace. The whole process was protected by argon gas.

### 2.3. Characterization Methods

The compositions of alloy samples were detected by inductively coupled plasma atomic emission spectrometry (Prodigy, Optima 4300 DV, Lehman, NY, USA). The compositions of slag and tailing samples were detected by Atomic absorption spectrophotometer (SU–Z2700, Tokyo, Japan). Tailing is the slag after desulfurization. C and S contents in slag and metal samples were detected by a carbon sulfur analyzer (G4 ICARUS, Bruker Ltd., Karlsruhe, Germany). The microstructures of alloy samples were determined by SEM (SU–8010, Hitachi, Tokyo, Japan), whose accelerating voltage and beam current were 20 kV and 20 µA, respectively. Thermodynamic calculations were carried out using FactSage (FactSage 7.5, Thermfact/CRCT and GTTTechnologies, Montreal and Aachen, Canada and Germany) with pure substance (FactPS), oxide (FToxid), alloy, and sulfide (FTmisc) databases [1,23].

## 3. Results and Discussion

### 3.1. Desulfurization by Fe–65 wt.% Mn

**Figure 4:**
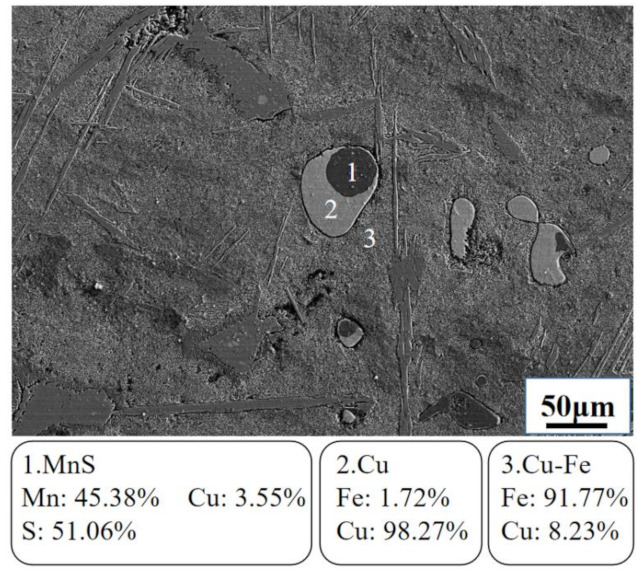
Electron microscope of Cu–Fe alloy after desulfurization by Fe–Mn.

Part of the Mn will be oxidized and part will enter the metal. Fe–Mn as a desulfurizer was added in excess. The ratio of experimental raw materials are as follows: 40 g Cu–Fe alloy, 34 g tailing, 15 g CaO, and 7.2 g Fe–65 wt.% Mn (the atomic ratio of Mn to S is 5). The obtained chemical analysis results of the alloy and tailing after desulfurization are shown in Table 2. The S content decreased from 1.23 wt.% to 0.24 wt.%; the desulfurization rate was 80.49%. Fe–Mn alloy had a good desulfurization ability. Figure 4 shows the SEM microscopic analysis of the alloy after desulfurization. It can be seen that the main matrix of the alloy is an iron-rich phase, and the copper spheres aggregate into a copper-rich phase. 

The solubility of copper is relatively high in iron, and with a decrease in temperature, the solubility of copper gradually decreases. Cu–Fe alloys exhibit a liquid with a metastable state. Depending on the alloy composition, the two liquid phases separate when the melt is supercooled below a certain temperature. After desulfurization, the Fe phase and the Cu phase still exist, while the Cu_2_S phase and the FeS phase disappear. In the copper-rich phase, it can be seen that there is a certain MnS phase. MnS has a lower Gibbs free energy and is more stable than Cu_2_S and FeS. Therefore, it is speculated that the Fe–Mn desulfurization reaction of copper-containing pig iron at 1698 K (1425 °C) can be described as the following equation:

Mn + Cu_2_S = MnS + 2Cu (1)

Mn + FeS = MnS + Fe(2)

**Figure 5:**
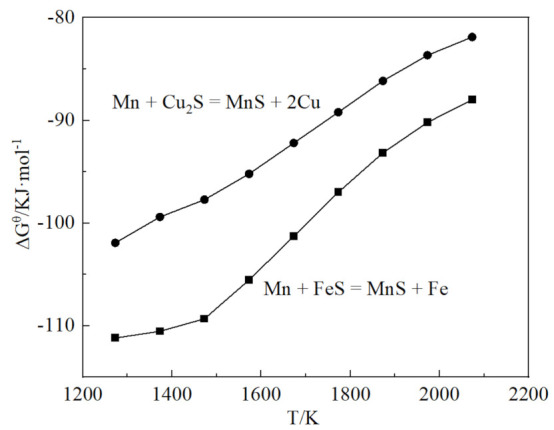
The Gibbs free energy of Mn desulfurization reaction.

Figure 5 shows the Gibbs free energy of the desulfurization reaction of Mn with Cu_2_S and FeS. The value of Gibbs free energy is negative. In this experiment, S reacted with Mn to form MnS, most of which entered the slag phase, while some of it was trapped in the matrix. The possible reason for this is that the low Fe–Mn addition is not enough to react with all the S present in the pig iron. With a low content of ferromanganese, there is not a sufficient desulfurization effect; therefore, we increased the amount of Fe–Mn to 30 g, from which the S content was reduced to 0.0013%, demonstrating a high desulfurization ability. 

### 3.2. Desulfurization by CaC_2_

The ratio of experimental raw materials is as follows: 40 g Cu–Fe alloy, 34 g tailing, 15 g CaO, and 10 g CaC_2_ (the atomic ratio of Ca to S is 8). The chemical analysis results of desulfurized Cu–Fe and tailing are shown in Table 3. It can be seen that the S content dropped to 0.0079%, indicating the good desulfurization performance of CaC_2_. The desulfurization of CaC_2_ in molten iron has also been studied by previous researchers [24,25]. They found that as the desulfurization reaction proceeded, a CaS layer with a thickness of about 120 μm was formed around CaC_2_, and a thin graphite layer was detected between the CaS layer and the remaining CaC_2_ particles. They believed that, under high-temperature conditions, CaC_2_ decomposes into calcium vapor and a layer of graphite. The calcium vapor reacts with sulfur in molten iron to form a layer of CaS and graphite. The graphite layer and CaS layer gradually thicken and form a barrier, which reduces the diffusion of calcium vapor and the progress of the desulfurization reaction. In the process of kinetic research, the desulfurization reaction of CaC_2_ was controlled by diffusion, and reducing the particle size of calcium carbide was helpful for the desulfurization reaction [25]. The reaction equation of CaC_2_ decomposition is as follows:

CaC_2_ = Ca + 2C (3)

∆G = ∆G^θ^ + RTln(P_Ca_/P^θ^) (4)

At different temperatures, calcium vapor has different partial pressures. As the reaction proceeds, the partial pressure of calcium vapor is reduced, and the reaction continues in the direction of generating calcium vapor, which promotes the decomposition of CaC_2_.

The reaction equation of CaC_2_ desulfurization is as follows:

CaC_2_ + Cu_2_S = CaS + 2Cu + 2C (5)

CaC_2_ + FeS = CaS + Fe + 2C (6)

Ca + Cu_2_S = CaS + 2Cu (7)

Ca + FeS = CaS + Fe(8)

**Figure 6:**
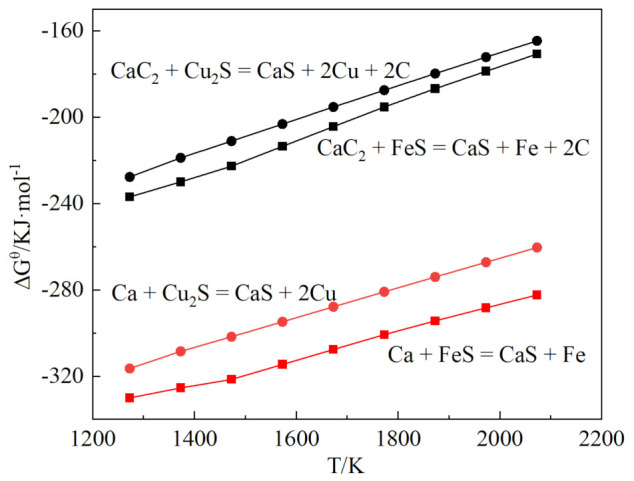
The Gibbs free energy of CaC_2_ desulfurization reaction.

**Figure 7:**
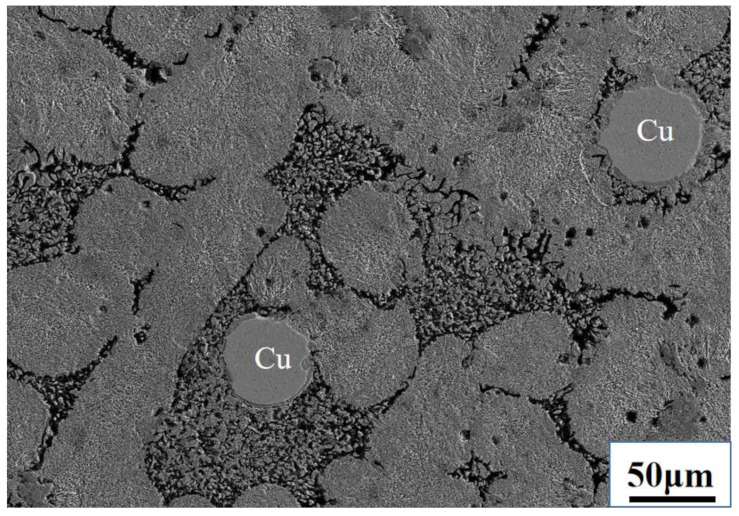
Electron microscope of Cu–Fe alloy after desulfurization by CaC_2._.

The Gibbs free energy of the desulfurization reaction of CaC_2_ is shown in Figure 6. CaC_2_ and Ca can react with FeS and Cu_2_S. Cu_2_S was relatively difficult to reduce. The SEM microstructure of the Cu–Fe alloy after CaC_2_ desulfurization is shown in Figure 7. The metal matrix is an iron-rich phase, which exists in the form of pearlite. A large amount of copper-rich phase is mixed in metal matrix, while the sulfur-containing phase disappeared. 

### 3.3. Effect of Desulfurization on the Existing Form of Copper in Alloy 

**Figure 8:**
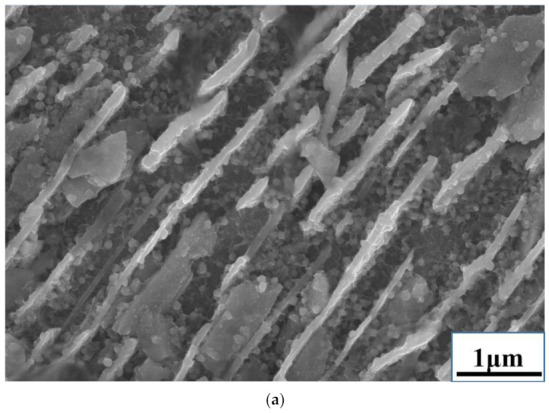

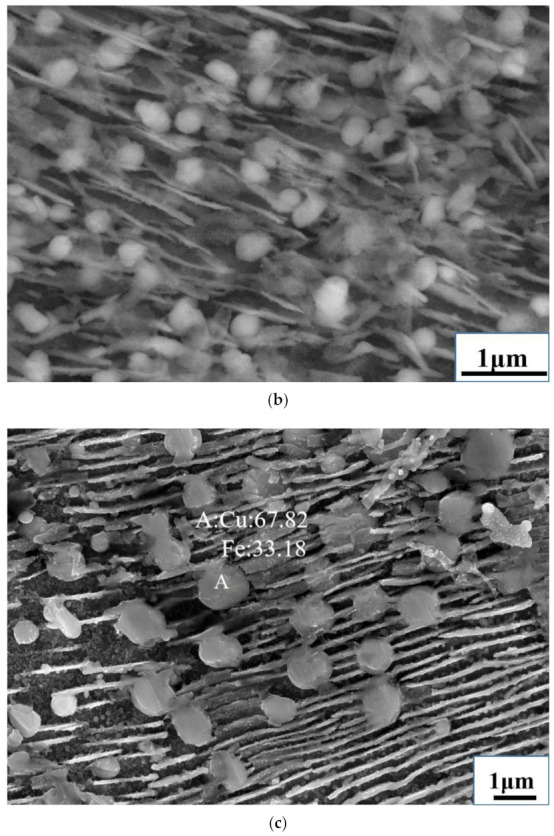
Nano-copper spheres in Cu−Fe (**a**) before desulfurization, (**b**) after desulfurization by Fe–Mn, and (**c**) after desulfurization by CaC_2_.

In addition, nano-copper spheres were found in the metal during the experiments. Nano-copper spheres dispersed in pearlite and on the walls of ferrite and cementite, which is typical image for nano-scale spherical particles. The results are shown in Figure 8. An EDS analysis of Cu–Fe alloy was carried out. The main component with a white spherical shape in Figure 8 was copper. Because the alloy was an iron-rich matrix, the point scan results show that there was also iron in the alloy. Before desulfurization, the diameter of nano-copper spheres was small, less than 100 nm. After desulfurization, there were still a large number of nano-copper spheres distributed in pearlite, but the diameter increased to 200–800 nm, and more along the wall of cementite. Due to the different melting points of copper and iron, iron will first solidify during the cooling process. When the liquid copper reaches equilibrium on the surface of the iron solid, the relationship between the contact angle and the interfacial tension conforms to Young’s equation. 

Perepezko et al. reported that, when the Fe content in Fe–Cu alloys exceeds 65 wt.%, these alloys and other phases cannot reach the critical point wetting conditions [26]. Salje and Fellerkniepmeier et al. measured the diffusion coefficient of Cu in Fe in the temperature range of 963–1323 K [27]. If nano-copper is formed during cooling, Cu spheres will grow, but liquid Cu cannot wet the surface of the Fe-rich phase due to solid diffusion; the Cu droplets contract and aggregate into spheres on the surface of the Fe phase. Zhang et al. studied the effect of S content on the contact angle of liquid slag and metal [28]. Sulfur in metal reduces the surface tension of molten iron and increases the contact angle, thereby affecting the diameter of the liquid copper. Therefore, a decrease in the sulfur content will promote an increase in the diameters of the nano-copper spheres. The nano-spheres provide obstacles for dislocation movement, and the size of nano-copper will have an influence the properties of Cu–Fe alloy.

## 4. Conclusions

The desulfurization process of Cu–Fe alloy obtained through the reduction of copper slag was studied. The conclusions are as follows:

(1) Sulfur exists in the form of Cu_2_S and FeS in Cu–Fe alloy, and CaO does not easily react with Cu_2_S. According to the thermodynamic calculation results, the Gibbs free energy values of the reaction of Mn and CaC_2_ with Cu_2_S are all negative, and the desulfurization reaction can be carried out.
(2) The addition of Fe–Mn and CaC_2_ could remove S from Cu–Fe alloy. When the addition of Fe–Mn made the atomic ratio of Mn to S 5, the S content was decreased to 0.24%. When the atomic ratio of Mn to S was 20.8, the S content was reduced to 0.0013%. When the atomic ratio of Ca to S was 8, the content of S was reduced to 0.0079%.
(3) The desulfurization reaction had an effect on the form of copper in the Cu-Fe alloy. Nano-sized copper spheres existed in the Cu–Fe alloy before and after desulfurization, and the spherical diameter became larger after desulfurization. The possible reason for this was that the liquid Cu could not wet the surface of the Fe-rich phase, and liquid copper shrank and aggregated into a spherical shape along the surface of the Fe-rich phase. As the S content decreased, the contact angle decreased and the size of nano-copper spheres expanded.

## Figures and Tables

**Table 1:** The composition of Cu–Fe alloy and slag after reduced (wt.%).

Composition	Fe	Cu	C	S	CaO	Al_2_O_3_	SiO_2_
Cu–Fe alloy	88.10	8.02	1.69	1.23	-	-	-
Slag after reduced	3.29	-	-	0.22	38.55	4.73	45.65

**Table 2:** The composition of Cu–Fe alloy and tailings after desulfurization by Fe–Mn (wt.%).

Composition	Mn	Cu	S	CaO	Al_2_O_3_	SiO_2_
Cu–Fe alloy	1.46	9.51	0.24	-	-	-
Tailings	7.84	-	0.90	55.10	3.14	30.43

**Table 3:** The composition of Cu–Fe alloy and tailings after desulfurization by CaC_2_ (wt.%).

Composition	Cu	S	Ca	Al_2_O_3_	SiO_2_
Cu–Fe alloy	7.85	0.0079	-	-	-
Tailings	-	0.95	43.87	2.67	25.87
